# Effects of Walking Direction and Cognitive Challenges on Gait in Persons with Multiple Sclerosis

**DOI:** 10.1155/2013/859323

**Published:** 2013-10-07

**Authors:** Douglas A. Wajda, Brian M. Sandroff, John H. Pula, Robert W. Motl, Jacob J. Sosnoff

**Affiliations:** ^1^Department of Kinesiology and Community Health, University of Illinois at Urbana-Champaign, 301 Freer Hall, 906 South Goodwin Avenue, Urbana, IL 61801, USA; ^2^University of Illinois College of Medicine at Peoria, 1 Illini Dr. Peoria, IL 61605, USA

## Abstract

Declines in walking performance are commonly seen when undergoing a concurrent cognitive task in persons with multiple sclerosis (MS). The purpose of this study was to determine the effect of walking direction and simultaneous cognitive task on the spatiotemporal gait parameters in persons with MS compared to healthy controls. Ten persons with MS (Median EDSS, 3.0) and ten healthy controls took part in this pilot study. Participants performed 4 walking trials at their self-selected comfortable pace. These trials included forward walking, forward walking with a cognitive task, backward walking, and backward walking with a cognitive task. Walking performance was indexed with measures of velocity, cadence, and stride length for each testing condition. The MS group walked slower with significantly reduced stride length compared to the control group. The novel observation of this investigation was that walking differences between persons with MS and healthy controls were greater during backward walking, and this effect was further highlighted during backward walking with added cognitive test. This raises the possibility that backward walking tests could be an effective way to examine walking difficulties in individuals with MS with relatively minimal walking impairment.

## 1. Introduction

Walking impairment is very common in persons with MS. Indeed, an estimated 85% of persons with MS report gait impairment as a major limitation [1]. Consequently, tests of walking performance are commonly used for identification and tracking of disease progression [2, 3]. Traditionally, walking impairment is clinically indexed by timed walking performance tests, such as the timed 25-foot walk test. Although performance tests are clinically feasible, there has been concern regarding their sensitivity to walking impairment, and this might unnecessarily delay gait rehabilitation [4]. 

There are several ways in which walking tests could improve their ability to distinguish between those with and without minimal walking impairment. For instance, there is evidence that the concurrent performance of a cognitive task results in differential worsening of walk performance in those with clinically isolated syndrome [5] and persons with MS [6] compared with healthy controls. Additionally, research in persons with Parkinson's disease has utilized backwards walking with and without added cognitive tasks as a method to perturb walking function [7, 8]. 

This pilot study examined the effect of walking direction and simultaneous cognitive task on spatiotemporal gait parameters in persons with MS compared to healthy controls. We hypothesized that differences in spatiotemporal parameters between persons with MS and controls would be greatest in backward walking while engaging in a concurrent cognitive task.

## 2. Methods

### 2.1. Participants

Ten persons with MS and ten healthy controls took part in this pilot study. Persons with MS were included if they had a neurologist-confirmed diagnosis of MS; the ability to walk independently without the use of a walking aid; understood written and spoken English; and were relapse free for 30 days. Healthy controls were chosen as a convenience sample of individuals with no known diagnosis of MS but who satisfied the other inclusion criteria.

### 2.2. Protocol

Upon arrival to the clinic, participants were reminded of study procedures and provided written informed consent. Participants then completed a demographics questionnaire and performed 4 walking trials at their self-selected pace (i.e., normal, comfortable walking pace). These trials included forward walking, forward walking while simultaneously performing a cognitive task, backward walking, and backward walking while simultaneously performing a cognitive task. Modified word list generation (WLG) was used as the simultaneous cognitive task for both forward walking and backward walking. Previous studies have utilized WLG to assess cognitive impairment in MS [9] and also to investigate the effects of cognitive motor interaction during walking in persons with MS [5, 10, 11]. Subjects were allowed to rest between trials. 

All walking trials were completed over a 10-meter course partially covered by an 8-meter GAITRite electronic walkway made of thin rubber with embedded sensors that record the spatial and temporal characteristics of footfalls during gait. For the walking trials, subjects were asked to start 1 meter in front of the mat and end the trial at least 1 meter past the end of the mat. All participants completed the walking trials in the following order: forward walking, forward walking-word list generation, backward walking, then backward walking-word list generation. A single practice trial for backward walking was completed before subjects completed the measured backward walking trials. For the word list generation trials, participants were instructed to verbally list out loud as many fruits and vegetables as possible (e.g., apple, bean, and kumquat) for the forward walking trial and as many states in the United States as possible (e.g., Alaska, Ohio, and Wyoming) during the backward walking trial. Performance on the WLG cognitive task during the dual task conditions was indexed by the number of words uttered during the trial normalized by ambulation time. For all completed trials, the GAITRite software calculated the subject's spatiotemporal gait parameters including velocity, cadence, and stride length. 

### 2.3. Statistical Analysis

All statistics were performed in SPSS version 20.0 (IBM, Inc., Chicago, IL). Descriptive statistics (mean ± standard deviation) were calculated for all of the demographic and spatiotemporal gait parameters of interest. An independent samples *t*-test was used to compare the average ages of the two samples. Additionally, a 2 × 2 repeated-measures analysis of variance (ANOVA) with group (MS versus Control) and walking direction (forward versus backward) as the factors was used to analyze performance on the WLG task. We used a 2 × 2 × 2 mixed model ANOVA to compare differences in the spatiotemporal gait parameters for the four walking conditions. The model included group (MS versus Control) as the between subjects factor in addition to walking direction (forward versus backward) and cognitive condition (no cognitive task versus simultaneous cognitive task) as within subjects factors. 

## 3. Results

### 3.1. Demographics

There was a significant difference in age between the groups with the MS group consisting of older participants compared to the healthy controls (54.3 ± 11.0 versus 34.4 ± 8.9, *t*(18) = 4.4, *P* < 0.01). The distribution of sex was not equivalent across groups as the MS group included more females than the healthy control group (90% versus 50%). For the MS group, the range of EDSS scores was 2.5–4.0, and the average time since diagnosis was 15.3 years (SD = 11.1).

An analysis of performance on the cognitive task while walking both forwards and backwards revealed an effect of group but not walking direction. On average the healthy controls uttered more words per second than the MS group (1.5 (SD = 0.3) versus 1.0 (SD = 0.3)). Neither group significantly changed their performance based on walking direction.

### 3.2. Gait Parameters

**Figure 1:**
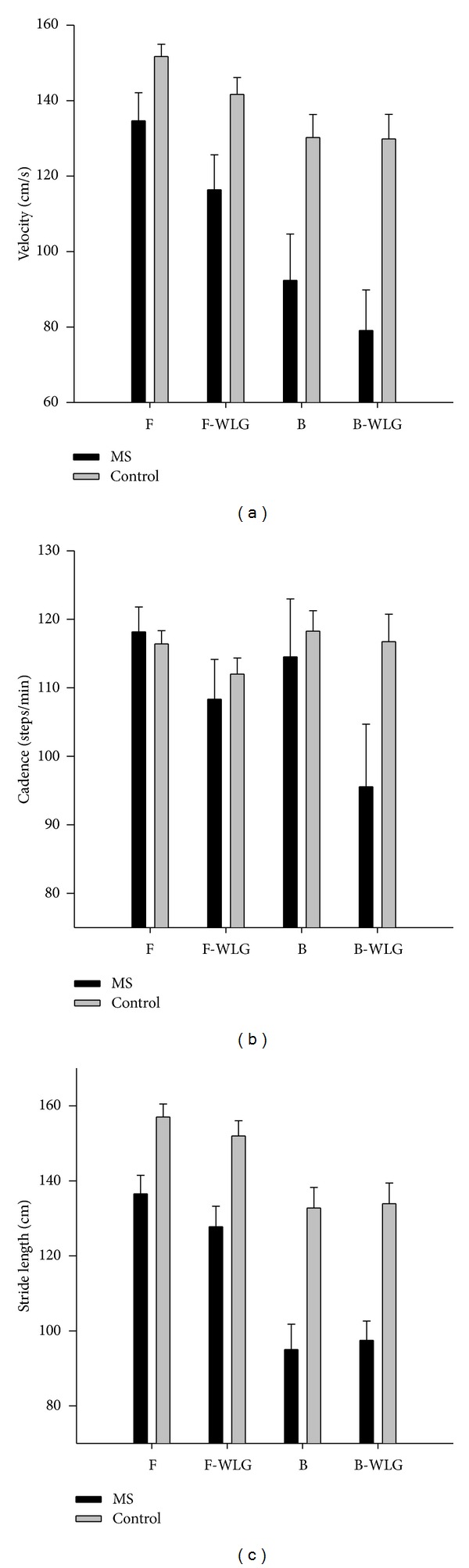
Walking velocity (a), cadence (b), and stride length (c) as a function of walking direction and group.

Mean ± standard error values of walking velocity, cadence, and stride length are presented in Figure 1 for each group and walking task. Results from the ANOVA including main effects and two-way interactions with estimated effect sizes are outlined in Table 1. Three-way interactions are visually depicted in Figures 1(a)–1(c).

### 3.3. Velocity

Overall, the MS group walked slower than the healthy controls. Both groups walked slower in the backwards direction compared to the forward direction. Similarly, all participants exhibited decreased walking velocity while performing a concurrent cognitive task compared to walking alone.

 Multiple interactions were observed for walking velocity (see Figure 1(a)). First, the interaction between group and direction resulted from the MS group having a greater decline in velocity from forwards to backwards walking. Likewise, the interaction between group and cognitive condition resulted from the MS group having greater decline in velocity in the dual task conditions than the healthy controls. Lastly, the interaction between direction and cognitive task stemmed from a greater reduction in velocity while performing the simultaneous cognitive task in the forward walking condition compared to backward walking when data is collapsed across groups.

### 3.4. Cadence

No significant differences in cadence were observed with respect to group or walking direction. All participants displayed reduced cadence when walking while performing the simultaneous cognitive task. Furthermore, an interaction between group and cognitive condition resulted from the MS group demonstrating a greater reduction of cadence under the cognitive task conditions compared to simple walking, whereas the healthy controls did not display this change. Finally, an interaction between group, walking direction, and cognitive condition was observed for cadence (*F* = 5.92, *P* = 0.03, *η* ^2^ = 0.25). This interaction resulted from a greater reduction of cadence in the backward walking direction compared to the forward walking direction with the additional cognitive task—only in the MS group (see Figure 1(b)). 

### 3.5. Stride Length

On average the MS group took shorter strides than the healthy controls, whereas all participants displayed shorter stride length under the backward walking conditions compared to forward walking. An interaction between group and walking direction was the result of the MS group having a greater reduction in stride length from forwards to backwards walking compared to the healthy controls. There was a significant interaction between walking direction and cognitive condition due to a reduction of stride length for all participants while completing the simultaneous cognitive task in the forward direction, whereas an increase in stride length was observed for participants performing the cognitive task during backward walking (see Figure 1(c)). 

## 4. Discussion

This study analyzed the effect of walking direction and simultaneous cognitive task performance on gait parameters in persons with MS and healthy controls. As expected, persons with MS walked slower with shorter strides than healthy controls. The novel observation of this investigation was that walking differences between persons with MS and healthy controls were greater during backward walking, and this effect was further highlighted during backward walking with an added cognitive test. This raises the possibility that backward walking tests could be an effective way to identify walking impairment in individuals with MS. 

 The difficulty of the walking tests was manipulated with two distinct manipulations: walking direction and concurrent cognitive tasks. Although backward walking has been utilized in other clinical populations [7, 8, 12], this is the first investigation that has used backward walking in persons with MS. From a control standpoint, an individual must rely on proprioception and vestibular function to successfully walk backwards. Given that both of these sensory systems are often impaired in persons with MS [13, 14], it is not surprising that backward walking performance in this sample of persons with MS was worse than that of healthy controls. It is also possible that the decrease in walking speed during backward walking observed in persons with MS might be attributed to a more cautious gait based on a compensatory mechanism to minimize the risk of falling [15]. Regardless of the possible mechanisms underlying group differences in backward walking, the observed results raise the possibility that backward walking tasks could be used to examine preclinical impairment in gait in persons with MS. 

The other experimental manipulation involved a concurrent cognitive task during the walking trials. Consistent with previous research [5, 6, 10], the MS group decreased their walking performance with the simultaneous performance of a cognitive task. The control group did not alter their walking performance with the concurrent cognitive task. Alterations in gait with a concurrent cognitive task are often explained within the context of the capacity model of attention, which suggests that there is a finite limit on a person's cognitive resources [16]. Under dual task conditions both the motor and cognitive tasks compete for these resources. Alterations of gait while performing a simultaneous cognitive task might be attributed to (1) limited cognitive resources; (2) greater cognitive requirement of walking; (3) deliberate prioritization of one task over the other; or (4) a combination of these factors. Seemingly, the observation that the MS group uttered fewer words during walking coincides with the capacity model of attention. 

As previously mentioned, the findings of this study indicate that more challenging tasks such as backward walking with and without a cognitive task could be beneficial as clinical tests. In terms of gait velocity, the more challenging backward walking with simultaneous cognitive task was able to better identify significant differences between the changes in gait of the MS and control groups. Additionally, performance on backward walking tests has recently been related to falls status in older adults [12]. Given the relationship between falls and gait impairment in persons with MS [17], it is possible that backward walking could provide a unique indicator of fall risk in this clinical population. Finally, these tests could possibly be adapted and implemented to the clinical setting with minimal effort using standard timing procedures. Future research utilizing backward walking tests with a larger sample and matched controls is warranted to determine the possible clinical capabilities of these tests. Ultimately, the ability to better identify mild gait impairment can lead to earlier intervention and rehabilitation for individuals with MS [4].

### 4.1. Study Limitations

This pilot study included multiple limitations which must be taken into account. The first limitation is the small sample size for both groups suggesting the importance of replication in a larger sample. A second limitation in regards to the sample was the lack of demographic matching between the control group and MS group, as the control group was significantly younger with a different gender distribution relative to the MS sample. Another limitation was the lack of randomization and counterbalancing of the ordering of the walking trials. Consequently, it is possible that performance on later trials was affected by fatigue, practice, and/or possible cognitive loading differences (i.e., phrasing states versus fruits). However, because the participants had relatively minimal gait impairment, the likelihood that they would be fatigued after a few relatively short walking tests is minimal. Additionally, no baseline data were recorded for the cognitive tasks without a walk. This information would be required to look into the possible effects of task prioritization on the calculated dual task changes of walking. Finally, no standard neuropsychological tests were administered to identify possible cognitive differences between the groups. 

## 5. Conclusions

 This study provided a novel examination of the effect of walking direction and simultaneous cognitive task on walking performance in persons with MS. The results indicate that walking differences between the MS group and healthy controls were the greatest when walking backwards. The findings also suggest that these more challenging walking tests could have greater sensitivity to identifying gait deficits in persons with MS who have minimal walking impairment. Furthermore, the results showed a possible distinction in the behavior of the groups based on walking direction. Further research should be carried out to identify the mechanisms contributing to gait declines in persons with MS and the usefulness of possible effect minimization techniques.

## Figures and Tables

**Table 1:** Main effects and two-way interactions for spatiotemporal gait parameters.

Main effects	Velocity (cm/s)				Cadence (steps/min)				Stride length (cm)			
	Mean ± SD	*F*	*P*	*η* ^2^	Mean ± SD	*F*	*P*	*η* ^2^	Mean ± SD	*F*	*P*	*η* ^2^
Group												
MS	**105.6** ± **37.5**	**10.8**	**0.00**	**0.37**	109.1 ± 23.3	1.3	0.27	0.07	**114.2** ± **25.2**	**19.9**	**<0.01**	**0.52**
Control	**138.4** ± **18.4**				115.9 ± 9.2				**143.9** ± **18.0**			
Direction												
Forward	**136.1** ± **23.8**	**36.0**	**<0.01**	**0.67**	113.7 ± 12.1	0.4	0.56	0.02	**143.3** ± **18.2**	**141.8**	**<0.01**	**0.89**
Backward	**107.9** ± **36.4**				111.3 ± 22.4				**114.8** ± **25.7**			
Cognitive condition												
None	**127.2** ± **32.7**	**25.5**	**<0.01**	**0.59**	**116.8** ± **15.1**	**14.5**	**<0.01**	**0.45**	130.3 ± 27.8	3.2	0.09	0.15
Dual	**116.7** ± **34.3**				**108.2** ± **19.6**				127.8 ± 25.2			
Group × direction												
MS forward	**125.5** ± **27.5**	**6.1**	**0.02**	**0.25**	113.3 ± 15.8	1.9	0.18	0.10	**132.1** ± **16.6**	**9.4**	**<0.01**	**0.34**
MS backward	**85.7** ± **36.1**				105.0 ± 28.8				**96.2** ± **18.5**			
Control forward	**146.7** ± **13.1**				114.2 ± 7.0				**154.5** ± **11.9**			
Control backward	**130.0** ± **19.4**				117.5 ± 10.9				**133.3** ± **16.9**			
Group × cognitive condition												
MS none	**113.5** ± **38.0**	**6.5**	**0.02**	**0.26**	**116.3** ± **20.1**	**6.3**	**0.02**	**0.26**	115.8 ± 28.0	0.2	0.68	0.01
MS dual	**97.7** ± **36.3**				**101.9** ± **24.5**				112.6 ± 22.5			
Control none	**141.0** ± **18.6**				**117.3** ± **7.8**				144.9 ± 18.8			
Control dual	**135.8** ± **18.2**				**114.4** ± **10.4**				142.9 ± 17.6			
Direction × cognitive condition												
Forward none	**143.2** ± **19.7**	**4.5**	**0.05**	**0.20**	117.3 ± 9.0	1.6	0.22	0.08	**146.8** ± **16.8**	**11.9**	**<0.01**	**0.40**
Forward dual	**129.0** ± **25.9**				110.2 ± 13.8				**139.8** ± **19.3**			
Backward none	**111.3** ± **35.6**				116.4 ± 19.6				**113.9** ± **27.1**			
Backward dual	**104.5** ± **37.7**				106.2 ± 24.3				**115.7** ± **24.9**			

Note: bolded values represent statistically significant effects.

## References

[1] LaRocca NG (2011). Impact of walking impairment in multiple sclerosis: perspectives of patients and care partners. *The Patient*.

[2] Bethoux F, Bennett S (2011). Evaluating walking in patients with multiple sclerosis. *International Journal of MS Care*.

[3] Motl RW (2013). Ambulation and multiple sclerosis. *Physical Medicine & Rehabilitation Clinics of North America*.

[4] Spain RI, St. George RJ, Salarian A (2012). Body-worn motion sensors detect balance and gait deficits in people with multiple sclerosis who have normal walking speed. *Gait and Posture*.

[5] Kalron A, Dvir Z, Achiron A (2010). Walking while talking—difficulties incurred during the initial stages of multiple sclerosis disease process. *Gait and Posture*.

[6] Hamilton F, Rochester L, Paul L, Rafferty D, O’Leary CP, Evans JJ (2009). Walking and talking: an investigation of cognitive-motor dual tasking in multiple sclerosis. *Multiple Sclerosis*.

[7] Hackney ME, Earhart GM (2009). Backward walking in Parkinson’s disease. *Movement Disorders*.

[8] Hackney ME, Earhart GM (2010). The effects of a secondary task on forward and backward walking in Parkinson's disease. *Neurorehabilitation and Neural Repair*.

[9] Boringa JB, Lazeron RHC, Reuling IEW (2001). The brief repeatable battery of neuropsychological tests: normative values allow application in multiple sclerosis clinical practice. *Multiple Sclerosis*.

[10] Sosnoff JJ, Boes MK, Sandroff BM, Socie MJ, Pula JH, Motl RW (2011). Walking and thinking in persons with multiple sclerosis who vary in disability. *Archives of Physical Medicine and Rehabilitation*.

[11] Sosnoff JJ, Socie MJ, Sandroff BM (2013). Mobility and cognitive correlates of dual task cost of walking in persons with multiple sclerosis. *Disability and Rehabilitation*.

[12] Fritz NE, Worstell AM, Kloos AD, Siles AB, White SE, Kegelmeyer DA (2013). Backward walking measures are sensitive to age-related changes in mobility and balance. *Gait & Posture*.

[13] Cattaneo D, Jonsdottir J (2009). Sensory impairments in quiet standing in subjects with multiple sclerosis. *Multiple Sclerosis*.

[14] Frohman EM (2003). Multiple sclerosis. *Medical Clinics of North America*.

[15] Matsuda PN, Shumway-Cook A, Ciol MA, Bombardier CH, Kartin DA (2012). Understanding falls in multiple sclerosis: association of mobility status, concerns about falling, and accumulated impairments. *Physical Therapy*.

[16] Kahneman D (1973). *Attention and Effort*.

[17] Cattaneo D, de Nuzzo C, Fascia T, Macalli M, Pisoni I, Cardini R (2002). Risks of falls in subjects with multiple sclerosis. *Archives of Physical Medicine and Rehabilitation*.

